# Use of oral health care services in Finnish adults – results from the cross-sectional Health 2000 and 2011 Surveys

**DOI:** 10.1186/s12903-017-0364-7

**Published:** 2017-04-24

**Authors:** Anna L. Suominen, Sari Helminen, Satu Lahti, Miira M. Vehkalahti, Matti Knuuttila, Sinikka Varsio, Anne Nordblad

**Affiliations:** 1 Institute of Dentistry, University of Eastern Finland, P.O. BOX 1627, 70211 Kuopio, Finland; 2 Department of Oral and Maxillofacial Surgery, Kuopio University Hospital, P.O. BOX 100, Kuopio, 70029 KYS Finland; 3 The Living Environment and Health Unit, National Institute for Health and Welfare (THL), P.O. BOX 95, 70701 Kuopio, Finland; 4 The Health Monitoring Unit, National Institute for Health and Welfare (THL), P.O. BOX 30, 00271 Helsinki, Finland; 5 The Social Insurance Institution (Kela), P.O. BOX 78, 00380 Helsinki, Finland; 6 Department of Community Dentistry, Institute of Dentistry, University of Turku, 20014 Turun yliopisto, Turku, Finland; 7 Department of Oral and Maxillofacial Diseases, Faculty of Medicine, University of Helsinki, P.O. BOX 41, 00014 Helsinki, Finland; 8 Medical Research Center, Oulu University Hospital & Oulu University, 90220 Oulu, Finland; 9 Department of Social Services and Health Care, City of Helsinki, P.O. BOX 6452, 00099 Helsinki, Finland; 10 Department of Social and Health Services, Unit of Health Services, The Ministry of Social Affairs and Health, PO Box 33, FI-00023 Government, Finland

**Keywords:** Health service use, Visits to a dentist, National survey, Health care reform, Subsidization, Cost of care

## Abstract

**Background:**

During the 2000s, two major legislative reforms concerning oral health care have been implemented in Finland. One entitled the whole population to subsidized care and the other regulated the timeframes of access to care. Our aim was, in a cross-sectional setting, to assess changes in and determinants of use of oral health care services before the first reform in 2000 and after both reforms in 2011.

**Methods:**

The data were part of the nationally representative Health 2000 and 2011 Surveys of adults aged ≥ 30 years and were gathered by interviews and questionnaires. The outcome was the use of oral health care services during the previous year. Determinants of use among the dentate were grouped according to Andersen’s model: predisposing (sex, age group), enabling (education, recall, dental fear, habitual use of services, household income, barriers of access to care), and need (perceived need, self-rated oral health, denture status). Chi square tests and logistic regression analyses were used for statistical evaluation.

**Results:**

No major changes or only a minor increase in overall use of oral health care services was seen between the study years. An exception were those belonging to oldest age group who clearly increased their use of services. Also, a significant increase in visiting a public sector dentist was observed, particularly in the age groups that became entitled to subsidized care in 2000. In the private sector, use of services decreased in younger age groups. Determinants for visiting a dentist, regardless of the service sector, remained relatively stable. Being a regular dental visitor was the most significant determinant for having visited a dentist during the previous year. Enabling factors, both organizational and individual, were emphasized. They seemed to enable service utilization particularly in the private sector.

**Conclusions:**

Overall changes in the use of oral health care services were relatively small, but in line with the goals set for the reform. Older persons increased use of services in both sectors, implying growing need. Differences between public and private sectors persisted, and recall, costs of care and socioeconomic factors steered choices between the sectors, sustaining inequity in access to care.

## Background

Health policies are expected to have an impact on population health and health inequalities. Organization of care is one of the tools to achieve these aims. In Finland, the state’s responsibility to promote welfare, health and security is rooted in the Constitution [1]. This enshrines the right of everyone to income and to care if they are unable to manage adequately. The duties of municipal authorities throughout Finland to arrange social and health care are stipulated by laws on social and health care planning and the central government transfers to local government. Finland is a sparsely populated country in the Northern Europe with 5,4 million inhabitants. In Finland, the average household net-adjusted disposable income per capita is USD 28 238 a year, less than the Organisation for Economic Co-operation and Development (OECD) average of USD 29 016 a year and 87% of adults aged 25–64 have completed upper secondary education, higher than the OECD average of 76% [2]. As a result of decades of post-war economic growth and reconstruction, Finland today represent a Nordic welfare state which offers its residents generous and universal health care oral health care services being an exception. Overall, the use of oral health care services has been on lower level in Finland compared to the other Nordic countries with similar health care systems, i.e., generous and universal welfare provision. One reason for the lower use of services has been the large number of edentulous persons in Finland who do not seek care [3]. Another reason has been the relatively high cost of care owing to limited access to publicly funded care. OECD findings have earlier shown clear income-related inequity in visits to a dentist in the late 1990s. Finland was among the most inequitable countries [4], which was also shown in another study [5]. Strategies to improve access to dental care for disadvantaged or underserviced populations include reducing financial and non-financial barriers. Therefore, a major oral health care reform was implemented in Finland in 2001–2002. The aim of this reform was to increase equity in access to care. According to the reasoning behind the reform, this would result in overall increase in the use of services and ultimately, in better oral health of the population.

In Finland, children and adolescents have been entitled to care free of charge in Public Dental Services (PDS) since the 1950s including necessary orthodontics (Table 1). Adults have mainly paid their care by themselves. However, for adults, publicly subsidized oral health care, either in the form of access to the PDS or partial reimbursement on private dentist’s fees from the National Sickness Insurance (NSI), has been gradually expanded. Since 1990, the subsidy system covered citizens born in 1956 or later. The latest reform was implemented in 2001–2002 since when the whole Finnish population has been entitled to the PDS or services have partly been reimbursed by the NSI.

**Table 1:**
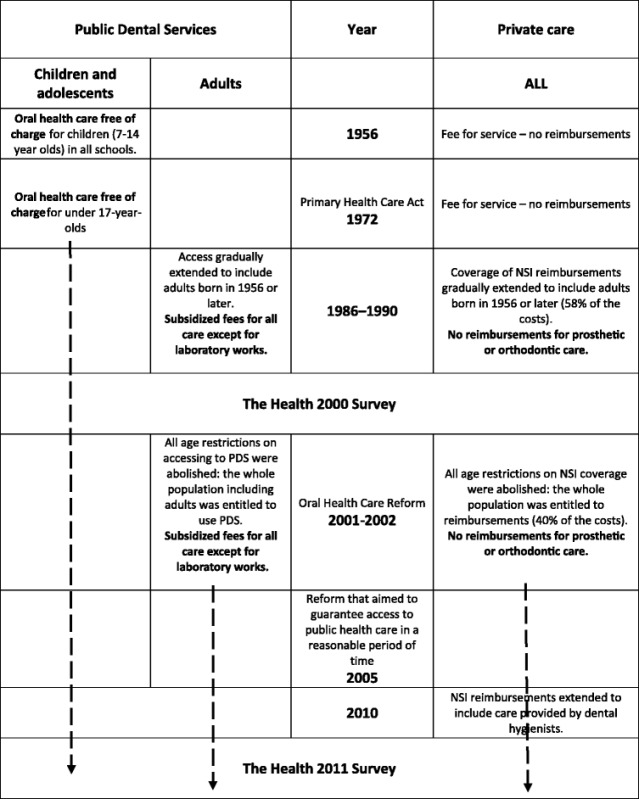
Summary of expansion of publicly funded oral health care in Finland in 1956–2011

After the subsidization reform in 2001–2002, the PDS were no longer allowed to discriminate adults by age in care provision, and the access to PDS had to be based on need. This and the reduced costs due to reimbursements for private care were supposed to increase demand in both service sectors, which are equally sized, based on the number of dentists working in them. However, the use of PDS is clearly cheaper from patients’ point of view. User charges in the PDS are lower than costs after reimbursements in the private sector. Reimbursement on private costs was in effect less than 40% of the actual cost for the patient just after the reform and 35% in 2011. In addition, the reimbursements in the private sector has never covered any prosthetic care nor costs of prosthetic laboratory work. In the PDS, prosthetic care was offered in subsidized prices excluding the laboratory work. Hence, it was anticipated that most of the new users of oral health care and those less well-off would seek care in the PDS. On the other hand, in Finland, adults in general, and more specifically, those who are better off, have visited private dentists due to the history of organization of dental care. Middle-aged and older Finnish adults have a lot of need for prosthetic dental care [6], and specialists in prosthetic care mainly work as private practitioners. Therefore, the switch to PDS may also have been smaller due to the (possibly) long-term relationship with their current dentist.

In addition, in March 2005, new legislation took effect in Finland stating that non-urgent treatment and examinations at PDS and hospitals must be provided within clearly defined timeframes. Oral health care was included in this reform, which now stands as an important basis for the oral health care services at PDS. According to the law, patients must be able to contact their health centre immediately by phone during office hours also concerning oral health care. The need for oral treatment is assessed during this initial contact by a health care professional, for example a dental nurse. Any treatment that is considered odontologically necessary must be provided within a reasonable timeframe, within 6 months at the latest. In general, the number of dentists in Finland has remained relatively unchanged since 2003 and been about the average when compared to other European Union (EU) member states [7]. However, access to oral health care has been impaired by a lack of dentists in remote parts of Finland. It has not been possible to fill all dentists’ positions, which has caused problems in the PDS. Moreover, the number of private practitioners is clearly lower in rural areas. Therefore, oral health care has been further developed by adjusting the division of tasks between professional groups in oral health care, i.e., between dentists and dental hygienist or nurses. In 2010, the Ministry of Education also decided to increase the number of dentists educated in Finland by reopening the dental school at the University of Eastern Finland.

The aim of this study was to assess the changes in and the determinants of the use of oral health care services before the subsidization reform in 2000 and after both reforms in 2011.

## Methods

The data used in this study are part of the national Health 2000 and 2011 Surveys organized by the National Institute for Health and Welfare (THL) [8–10]. The Health 2000 Survey conducted in 2000-01 was a national survey of the Finnish population including adults aged 18 years and over living in mainland Finland. The main sample (8028) consisted of those aged 30 years and over and was recruited via stratified two-stage cluster sampling. Of those alive (7979), a total of 7415 (93%) participated in at least one part of the study and 7087 subjects (89%) were interviewed prior to the health examination or during the home health examination. The Health 2011 Survey was a follow-up study of the Health 2000 Survey. All participants of the Health 2000 Survey who were alive and living in Finland, excluding those denied, were invited. The sample of those aged 30 or over in 2011 consisted of 7964 adults, of whom 5806 (73%) participated in at least one part of the study, 5043 (63%) were interviewed during the health examination, home health examination or by phone, and 763 (10%) filled in a questionnaire afterwards. THL as the responsible organization of the surveys has according to the Medical Research Act (488/1999) applied and received an opinion in favour from the Ethics Committee for Epidemiology and Public Health of the Hospital District of Helsinki and Uusimaa, Finland both for the Health 2000 and 2011 Surveys. All participants in the both surveys have given their written informed consent. According to the Medical Research Act (488/1999), the permitted opinion in favour and informed consents cover the present study which the executive group of the Health 2000 and 2011 Surveys at the THL as an institutional review board (IRB) has approved.

In the Health 2000 Survey, a response on the use of oral health care services during the previous year according to a treatment place (PDS, private or other dentist, dental technician or dental hygienist) was received from 7067 participants. Of them, 5656 reported to be dentate, and of them, 5653 answered questions about the use of oral health care services. Participants were first asked ‘During the previous 12 months, how many times have you visited 1) a public sector dentist, 2) a private dentist, 3) some other dentist (Finnish Student Health Services (FSHS), Defence Forces, university clinic, hospital etc.), 4) a dental technician, or 5) received some other dental care’. Those who reported any visits to options ‘1–5’, were defined as having visited any oral health care during the previous year. Accordingly, those who reported any visits to options ‘1–3’ as having visited a dentist, those who reported any visits to option ‘1’ as having visited a PDS dentist, and those who reported any visits to option ‘2’ as having visited a private dentist during the previous year.

In the Health 2011 Survey, participants who attended the interviews or the questionnaire were asked ‘When did you last visit oral health care?’ with the answering options 1) during the previous 12 months, 2) 1 to 2 years ago, 3) 3 to 5 years ago, 4) over 5 years ago, or 5) I have never been to oral health care. Those who chose option ‘1’ were defined as having visited oral health care during the previous year. Responses were received from 5692 participants, of whom 4138 reported being dentate. The number of visits during the previous year according to service sector was asked only in the interview conducted during the health examination from 4664 participants. Of them, an answer was obtained from 4082 participants, of whom 3765 reported being dentate. Those who reported any visits to a PDS dentist, a private dentist or some other dentists (FSHS, Defence Forces, university clinic, hospital etc.) were defined as having visited a dentist. Accordingly, those who reported any visit to a PDS or a private dentist were defined as PDS or a private dentist visitor.

To study the determinants of visits to a dentist among the dentate participants, the Andersen’s behavioural model [11] were used. Dentate participants were determined by the question ‘Do you wear removable dental prostheses?’ with the answering options 1) full prostheses, no natural teeth 2) prostheses with natural teeth, 3) no prostheses and being dentate, and 4) no prostheses or natural teeth. Those having answered ‘2’ or ‘3’ were defined as dentate. The **predisposing** factors included sex and age group according to entitlement to subsidized care (30–44, 45–54, 55–64, or 65 years or older) before the subsidization reform. The **enabling** factors included educational level, recall, dental fear, habitual use of services, household income in the Health 2000 Survey and barriers of access to care in the Health 2011 Survey. Education was categorized into three classes: basic, intermediate and higher education. Those with no formal vocational training or upper secondary education were classified as having basic education; those who had completed vocational training or passed the matriculation examination were classified into the intermediate level of education, and those with higher education had degrees or diplomas from higher vocational institutions, polytechnics and universities. Recall was asked with the question ‘How did you make your most recent dental appointment?’ with the answering options 1) by oneself, 2) received an invitation, 3) made the next appointment already during the previous treatment period, 4) went to see a dentist without making an appointment. Those who chose option ‘2’ were defined as recalled. Dental fear was asked with the question ‘Do you find visits to a dentist frightening’ with the answering options 1) not at all, 2) more or less, or 3) very much. Those having chosen options ‘2’ or ‘3’ were defined as having dental fear. Habitual use of dental care was asked only from dentate participants with the question ‘Do you usually go to see a dentist?’ with the answering options 1) regularly for check-up, 2) only when having tooth ache or some other trouble, or 3) never. Those having chosen option ‘1’ were defined as having regular check-ups. In the Health 2000 Survey, monthly household income was asked during the health interview and then formatted into OECD equivalence scale, which allocates a weight of 1 to the first household member, 0.7 to each additional adult, and 0.5 to each child under 18 years old [12] The OECD income was categorized into lowest, middle, and highest. In the Health 2011 Survey, participants were asked if the following circumstances prevented them from getting the dentist’s treatment they want: 1) waiting lists, 2) poor connections to the place of treatment (e.g. health centre or hospital), and 3) excessively high service charges and prices (e.g. health centre and outpatient clinic fees) with the answering option yes or no? **Need** factors included perceived current need for dental care, self-rated oral health, and use of removable dentures. Current need was inquired with the question ‘Do you think you need dental care?’ and those who answered ‘yes’ were defined as having current need. Self-rated oral health was asked with the question ‘Is your current oral health?’ 1) good, 2) fairly good, 3) average, 4) fairly poor or 5) poor. Options ‘1’ and ‘2’ were combined and defined as good self-rated oral health and option ‘3–5’ as average/poor. Use of removable dentures were inquired with the same question that separated the dentate. Answering option ‘2’ indicated the denture wearers and option ‘3’ those not wearing dentures.

Statistical differences in prevalences of use between the years 2000 and 2011 were examined using chi square tests. Separate multivariate logistic regression analyses were used to study the association between visits to any dentist, a PDS or a private dentist during the previous year and the determining factors. Having any visits to a dentists, to a PDS dentist or to a private sector dentist served as outcomes and independent factors included the **predisposing**, **enabling** and **need** factors listed previously. Significance level was set to 0.05. Before running the analyses, Spearman rank correlations between the factors were checked and found to be low enough (the highest between age group and denture status in 2000 *r* = −0.428 and in 2011 *r* = −0.396). The data were weighted using inverse probability weighting to match the population sizes in different clusters and to form a nationally representative data set of adult Finns aged 30 years or older in both study years. The data analyses were performed using SAS Callable SUDAAN software to take into account the two-stage cluster sampling design and weightings.

## Results

Response rate for those interviewed and having answered on the use of oral health care service questions was 88% in the Health 2000 Survey and 71% in the Health 2011 Survey.

### Use of oral health care services during the previous year

The proportion of those having used **any oral health care services** (i.e., visited a dentist, a dental hygienist, a dental nurse or a dental technician) during the previous year increased slightly between the Health 2000 and Health 2011 Surveys (Table 2). An increase in the proportion of those who had visited **a dentist** was also seen among all participants, but not among the dentate. Except for visits to **a PDS dentist**, the increase in the use of **all oral health care** was highest among those aged 65 years or older. In 2000, the proportion of visitors in this age group had been clearly lowest, but in 2011 the differences between the age groups had levelled out. At the same time, the proportion of visitors decreased in the youngest age groups. These same trends were seen among the dentate. Women reported using **any oral health care services** (58% in 2000 and 56% in 2011) more often than men (51% in 2000 and 54% in 2011, *p* < 0.001 between the sexes in both years). The same goes for visits to **a dentist** (56% in 2000 and 60% in 2011, 48% in 2000 and 53% in 2011, *p* < 0.001, respectively). In 2011, one fifth of the participants reported visits to **a dental hygienist**, without any differences between the age groups.

**Table 2:**
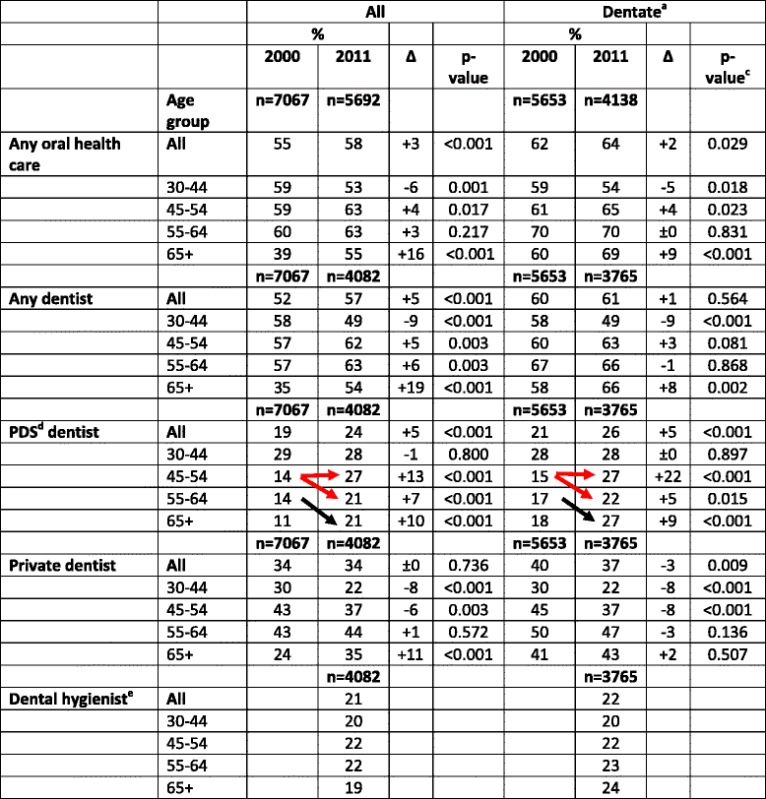
Proportions (%) of participants^a^ having visited oral health care^b^ in 2000 and 2011

^a^ Finnish adults aged ≥ 30 years ^b^ during the previous year, based on interview, ^c^ based on chi square, ^d^ Public Dental Service, ^e^ not available in 2000. The horizontal arrows indicate the period effect (difference between the same age groups in different years) and the diagonal arrow the ageing effect (the age group gets 10 years older)

In both surveys, a larger proportion of the participants reported visiting **a private dentist** than **a PDS dentist**. However, the differences had decreased, and the increased proportion of visitors to **any dentist** resulted from an increased use of **PDS dentists**. In the **private sector**, no change or decrease among the dentate participants was observed between the study years. The increase in **PDS use** was highest among those aged 44–54 years old, i.e., the first age group that became entitled to subsidized care after the Health 2000 Survey. The result is the same whether comparing the participants in this age group in the Health 2000 Survey to the same age group in the Health 2011 Survey (change almost doubled) or those being 10 years older in 2011 (change 50%). The first change was even more pronounced among the dentate. (Table 2) Dentate women visited both **PDS and private dentists** slightly more often than men and the changes in both sexes between the surveys were very similar in all age groups (Table 3).

**Table 3:**
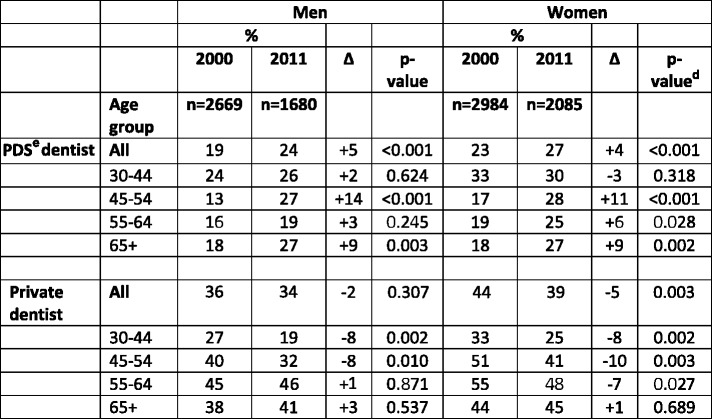
Proportions (%) of ***dentate*** ^a^ participants^b^ having visited a dentist^c^ in 2000 and 2011

^a^ based on interview, ^b^ Finnish adults aged ≥ 30 years, ^c^ during the previous year, based on interview, ^d^ based on chi square, ^e^ Public Dental Service

### Determinants for having visited a dentist during the previous year

Those with higher income visited **any**, and especially **a private dentist**, more often than those with lower income, who had more often visited **a PDS dentist** in 2000. The same goes for educational level, but these differences decreased between the surveys, the **PDS** being an exception. Those reporting regular check-ups or having been recalled had more often visited **a dentist**, especially **a private dentist** (Table 4).

**Table 4:**
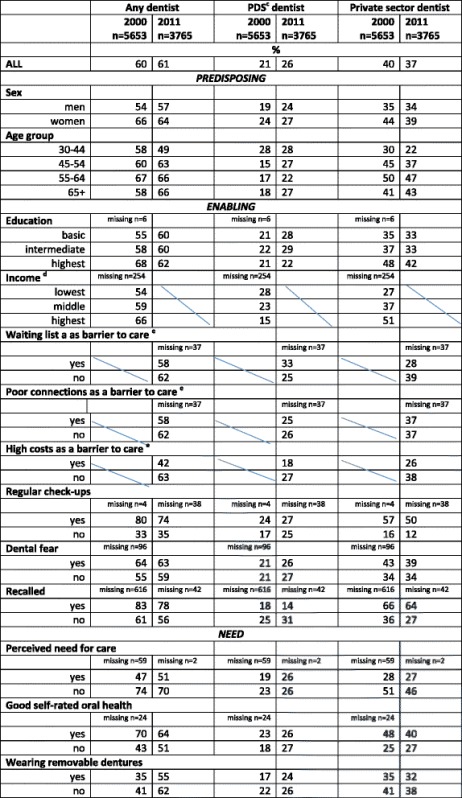
Proportions (%) of ***dentate*** ^a^ visitors^b^ in 2000 and 2011 by predisposing, enabling and need factors

^a^ based on interview, ^b^ Finnish adults aged ≥ 30 years having visited a dentist during the previous year, based on interview, ^c^ Public Dental Service, ^d^ available only in 2000, ^e^ available only in 2011

Self-reported regular dental check-ups were the most significant factor determining visits to **any dentist** in both surveys (Table 5). In addition, being female, recalled or reporting dental fear in both years increased the odds of dental visits. In the Health 2011 Survey, waiting lists to care in 2011 were reported to slightly increase the probability to visit **any dentist** during the past year. In contrast, perceived need for care decreased the probability to visit **any dentist** in both surveys, while younger age and middle income in the Health 2000 Survey and high costs of care in the Health 2011 Survey decreased the probability.

**Table 5:**
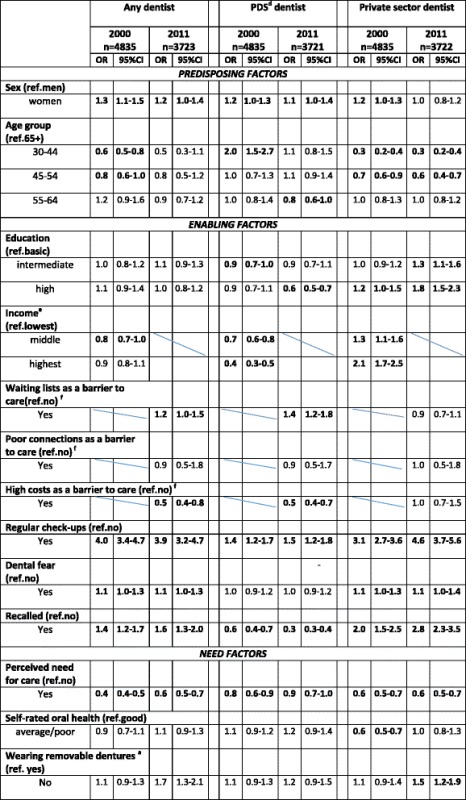
Adjusted ORs with 95%CI^a^ for having visited dentist^b^ among ***dentate*** ^c^ in 2000 and 2011

^a^ *OR* Odds ratio, *95%CI* 95% Confidence Interval (statistically significant in bold), *95%CI:s* 95%, ^b^during the previous year, ^c^Finnish adults aged ≥ 30 years, dentition status based on interview, ^d^ Public Dental Service, ^e^ available only in 2000, ^f^ available only in 2011

Being female, belonging to the youngest age group, or having regular, self-reported dental check-outs significantly increased the probability to visit **a PDS dentist** in the Health 2000 Survey. Having higher or middle educational level, being recalled by a dentist or having a perceived need for care decreased the odds. In the Health 2011 Survey, the result was the same except for the age group, which turned out to be insignificant (Table 5).

In the **private sector**, having regular, self-reported dental check-ups or being recalled by a dentist were most significantly associated with visits to a dentist in the Health 2000 Survey. In addition, being female and having higher or middle educational level increased the probability while younger age, perceived need for care or poorer self-rated oral health decreased the probability. In the Health 2011 Survey, sex or self-rated oral health were no longer significantly associated with visits, but having dental fear or no removable dentures increased the probability to visit **a private dentist**. The association between visits and having regular, self-reported check-ups or recall was even more pronounced (Table 5).

## Discussion

No major changes or only a minor increase in the overall use of oral health care services was seen between the study years. An exception were those belonging to the oldest age group who clearly increased their use of services. Also, a significant increase in visiting a PDS dentist was observed, particularly in the age groups that became entitled to subsidized care after the oral health care reform. In the private sector, the use of services decreased in younger age groups. The determinants for visiting a dentist, regardless the service sector, remained relatively stable; however, still with emphasis on the importance of the enabling factors. Being a regular dental visitor was the most significant determinant for having visited a dentist during the previous year. Younger age and waiting list increased the probability of visiting a PDS dentist while high education, income and the cost of care seemed to decrease the odds of PDS care. In the private sector, recall and regular check-ups were emphasized in addition to higher education and income.

A population-based sample and a response rate that was an exceptionally high in the Health 2000 Survey and high in the Health 2011 Survey are clear strengths of this study. However, the effects of non-response were larger in the Health 2011 Survey compared to the Health 2000 Survey. Lowest participation rates were seen among youngest men. Use of weights in analyses correct effects of non-response. These weights were in 2000 were based on age, sex, living area and mother language and in 2011 age, sex, education, physical activity, use of alcohol, use of vegetables, size of household and body mass index [10]. According to Härkänen et al. [13] statistical methods based on weighting or multiple imputation provide quite accurate results when comparing the true prevalence rates of disability pensions, hospitalization or reimbursements of medication obtained from national registers with the responses of the participants. Although these results alleviate the general concern of that non-participation creates biased results, low response rates in any survey are a true problem today. Those who did not attend the survey are probably persons who had not visited a dentist, and thus the results in 2011 may overestimate the use of services. Moreover, those who do not attend are also those in greatest need of care which mean that we lack information of those least well-off. While the repeated cross-sectional design does not allow any causal inferences, it does give comparable population-based results from two interesting occasions, i.e., before and after a major oral health care reform. The time between the two measurement points was on average 11 years and the time elapsed since the reform was 9 years, which is presumed to be sufficiently long to study the long-term effects of the reform. With regard to the measures employed in this study, recall bias is always a concern. However, all these measures are widely used and accepted in health service research. Yet, visiting during the previous year may already be a measure behind the times. Instead of the 1-year interval, visiting during the previous 2 years should be used, because need-based individual recall intervals even longer than 1 year have been a recommendation in Finland already since beginning of the 1990s and emphasized since the reform. This must have an impact on the average probability of dentist visits and probably partly explains the modest increase in the use of services during the previous year. In addition, European Union Statistics on Income and Living Conditions survey (EU-SILC) has suggested a new indicator to ascertain the quality of care with respect to unmet needs for dental examination [14]. This indicator can help countries monitor development and trends and determine the main characteristics of people with unmet needs. EU-SILC showed that unmet needs for dental examination vary among Nordic countries, and they also vary according to age, sex, income, education and activity status. In 2013, close to 8% of the EU-28 population had an unmet need for a dental examination or treatment, most of which were related to the organization of health services [15]. Being too expensive was by far the most common reason. After expense, the next most common reasons for unmet needs were fear (of dentists, hospitals, examination or treatment) and lack of time. Less common reasons were waiting to see if the problem resolved by itself, waiting lists, that it was too far to travel, or that the person did not know a good dentist or specialist. Exceptionally, a waiting list hindering a dental examination or treatment was the most frequent reason given in Finland.

Our results were similar to another Finnish study where data were gathered by repeated postal questionnaires in 2001, 2004 and 2007 [16]. However, in our study, the last measurement point was 4 years later than in the questionnaire study, thus giving a more up-to-date picture. The proportion of those who visited oral health care or a dentist during the previous year was a few percentage points lower in our study. The same is seen in comparison to other questionnaire surveys among working-age adults (aged 16–54 years) [17] or older persons (aged 65–84 years) [18]. These are probably due to different research contexts or methods used. The questionnaire surveys in 2001, 2004 and 2007 were specifically designed to study the effects of the oral health care reform, and the Health 200 and 2011 Survey participants were interviewed prior to or during a comprehensive clinical examination. Nevertheless, the increasing trends in demand for any oral health care or visiting a dentist were very similar.

Differences in methods, target populations or measures of use of services complicate comparisons between studies and countries. There are substantial differences in oral health care utilization rates of older persons (aged 50 years or over) across different European countries. For example, almost 82% of Swedish respondents but only about 24% of Polish respondents report having seen a dentist within the past year. In comparison with other European regions, there is a tendency toward more frequent and preventive dental treatment of the elderly populations residing in Scandinavia and Western Europe. Such utilization patterns appear only partially attributable to differences in the need for and accessibility of dental care [19].

The reform concerning the reduction of out-of-pocket expenses in dental care was implemented in 2001 and 2002. As a result, all age groups were entitled to cost-sharing, implying that part of the costs were paid out of public funds. According to the theory of demand for health [20], the reform was assumed to have its main impact on dental care use through the reduced out-of-pocket cost to the service users. Previous research has shown that demand for oral health care services increases when out-of-pocket costs are reduced by lowering charges or by means of subsidization or health insurance [21–27]. However, the present reform induced overall use only modestly. One could argue that this increase is wholly attributable to a decrease of edentulous persons (from 16 to 8%). Only those aged 30–44 years decreased their use, probably reflecting their good oral health but also grown demand of the older age groups. These kind of age and birth cohort effects in the demand for dental care due to improved dental health of the younger generations together with a decrease of edentulous persons in the older generations was reported already earlier. We, however, suggest that the results of the present study clearly showed an effect of the reform. Regardless of the service sector, the use of all oral health care increased significantly in the oldest age group (aged 65 years or over), implying not only that there were fever edentulous people but also a growing need for dental care among the elderly and their easier access to less costly care, also because of the differences shown between the PDS and private sector. In addition to the overall price effect, the reform opened access to PDS to all age groups, and therefore, large population groups were for the first time facing the choice between PDS and private dental service. Entitlement to subsidized care clearly increased the use of services in the PDS, which can partly be explained by lower fees, but also by a limited supply of private care outside larger cities. A decrease in use was shown particularly in the private sector in those aged 30–54 who have probably sought care in the PDS, if any. The overall effect on the private sector was non-existent or small, possibly due to high costs of care even after reimbursements, but also to lower supply of private services in remote areas. One obvious reason is also the qualified nature of the reform, i.e., exclusion of prosthetic care. It can also be an indication of the long-term dentist–patient relationships that are common in dentistry, but also of strong preferences (e.g. perceived quality) for one type of service, as shown in a study of a hypothetical choice between private and public dentist [28].

Regular, self-reported check-ups were the most significant determinant for visiting a dentist. This habit is not a custom among all Finnish adults since about 60% of those aged 30 years or over reported having regular check-ups in the Health 2000 Survey [6]. This is clearly lower than, for example, in Sweden, where 90% of adults claimed to be regular dental attenders [29]. Regarding determinants of care seeking, significant differences between service sectors were shown in this study. Regular check-ups and recall as a determinant for use of services were emphasized in the private sector. This was shown already in the 1990s, when recall turned out to be a central determinant of care-seeking [30, 31] and was significantly related to income and unemployment. Insufficient public availability and recall positively affected the choice of a private dentist, whereas income and dentist density increased the number of private visits [31]. These results are in line with the findings of the present study. It seems that the reform has improved overall access to care by lowering co-payments and user fees and increasing the public supply of dental care, but at the same time, due to the combined effects of efficient recall in the private sector, education, income and costs of care, socioeconomic inequalities in the use of services persist in Finland and impact the choice of the service sector. Interaction between these factors is complex, which was also shown in Germany, where the effects of income on the habitual use of dental care did not increase from 1989 to 2005 in spite of higher co-payments [32]. However, with regard to regular check-ups and recall, the results between the sectors or surveys are not fully comparable. In the PDS, recall for the whole population has never been the practice. Before the reform, only some special groups such as children, pregnant women or disabled persons were regularly invited to care. In 2011, when the whole population became entitled to subsidized care, the situation was different, but the patient themselves have been authorized to follow individualized check-up intervals defined by a dentist. The recall mechanisms in the private sector have been totally different. Overall, enabling factors, both organizational and individual, were emphasized in determining the use of oral health care services in both sectors. This is complemented by the associations detected between age groups and visits to dentists. This also indicates the effect of the reforms. Age is usually considered as a predisposing factor, but in this study it could also be an enabling factor since the age groups were categorized according to entitlement to subsidized care. Even though dental fear did show a significant role in this study, it can have a mediating role since it has previously been reported to be a major cause (41%) for irregular use of services among Finnish adults [33]. It is also not clear what are the effects of division of tasks between oral health care professionals. In 2007, the mean population to dentist ratio was 1500:1 and the mean population to dental hygienist ratio was 13,500:1 in the European Union/European Economic Area (EU/EEA) member states. In Finland, the respective figures were 1200:1 and 3000:1, being thus clearly below the mean [34]. Since then, the number of dental hygienists has grown steadily. According to findings of the Health 2011 Survey, a significant proportion of the participants had visited a dental hygienist during the past year. Unfortunately, comparable figures were not available from the Health 2000 Survey. However, it is probable that the increased use of oral health care service is also partly due to the increased number of visits to dental hygienists. The latter is shown also in statistics of the Social Insurance Institution (Kela) [35] but concerns only the private sector. Cost-effective division of tasks has long been a target in Finland and it seems to have begun to be put into place.

Recent OECD findings show that high-income persons are still more likely to have visited a dentist within the previous year, regardless of differences in public or private dental coverage and the amount of reimbursements. In this report, Finland was found among the average for the probability of a dental visit [36]. According to Eurostat [15], the frequency of reporting unmet needs for dental care for reasons of expense also decreased with increasing income and educational attainment in 2013. In a recent study based on World Health Surveys with country-level data, it was also suggested that more equal countries with regard to income have greater use of dental services [37]. According to another recent study based on cross-sectional analysis of data including older adults from 11 countries, dental systems relying on public coverage lower inequalities in their use, thus confirming the potential benefits of such systems [38]. However, the results from this study were commented as unlikely to provide strong evidence–further evidence is still needed to better understand how policy programs reduce inequalities in oral health and care [39]. According to the behavioural model of health service use, people’s use of health services is a function of individual and contextual characteristics [11], e.g., never solely due to organization or costs of care, but rather to a complex network including the structure of society and other social determinants. This may be the reason for the observation that oral health inequalities in the Scandinavian countries are very similar to inequalities in other European welfare state regimes [40].

It not clear what effect the decreased reimbursement level in the private sector (from 35 to 25% between years 2011 and 2015) has had on the use of services. Even more unclear is what will happen to oral health care services and oral health during the next large reform involving the whole social and health care sector in Finland. However, new measures of use which take into account the individually determined recall intervals and unmet need are needed in the future. These could include use of services during past 2 years instead of one or completed treatment periods according to need during past 3 years. Diverse and multidisciplinary research methods including qualitative approach are also warranted.

## Conclusions

The overall changes in the use of oral health care services were relatively small, but they were in line with the goals set for the reforms. Expansion of the PDS seemed to have larger effect than subsidizes in the private sector. However, significant changes by age groups were detected. Older persons increased their use of services regardless of the service sector, implying a growing need for dental care in the elderly. The differences between the public and private sectors persisted, and recall, costs of care and socioeconomic factors steered the choice between the sectors, sustaining inequity in access to care.

## Abbreviations

EU: European Union
EU/EEA: European Union/European Economic Area
EU-28: Present EU Member States
EU-SILC: European Union Statistics on Income and Living Conditions
FSHS: Finnish Student Health Services
Kela: Social Insurance Institution
NSI: National Sickness Insurance
OECD: Organisation for Economic Co-operation and Development
PDS: Public Dental Services
THL: National Institute for Health and Welfare
